# Self-Healing Liquid Metal Magnetic Hydrogels for Smart Feedback Sensors and High-Performance Electromagnetic Shielding

**DOI:** 10.1007/s40820-023-01043-3

**Published:** 2023-03-31

**Authors:** Biao Zhao, Zhongyi Bai, Hualiang Lv, Zhikai Yan, Yiqian Du, Xiaoqin Guo, Jincang Zhang, Limin Wu, Jiushuai Deng, David Wei Zhang, Renchao Che

**Affiliations:** 1 School of Microelectronics, Fudan University, Shanghai, 2000433 People’s Republic of China; 2 Laboratory of Advanced Materials, Shanghai Key Lab of Molecular Catalysis and Innovative Materials, Academy for Engineering & Technology, Fudan University, Shanghai, 200438 People’s Republic of China; 3 Key Laboratory of Separation and Processing of Symbiotic-Associated Mineral Resources in Non-Ferrous Metal Industry, School of Chemical & Environmental Engineering, China University of Mining & Technology (Beijing), Beijing, 100083 People’s Republic of China; 4 Institute of Optoelectronics, Fudan University, Shanghai, 200433 People’s Republic of China; 5 Henan Key Laboratory of Aeronautical Materials and Application Technology,, School of Material Science and Engineering, Zhengzhou University of Aeronautics, Zhengzhou, 450046 Henan People’s Republic of China; 6 Zhejiang Laboratory, Hangzhou, 311100 People’s Republic of China; 7 Inner Mongolia University, Hohhot, 010021 People’s Republic of China

**Keywords:** EMI shielding, Liquid metal, Hydrogel, Self-healing properties, Strain sensor, Magnetic patterning

## Abstract

Hydrogels exhibit potential applications in smart wearable devices because of their exceptional sensitivity to various external stimuli. However, their applications are limited by challenges in terms of issues in biocompatibility, custom shape, and self-healing. Herein, a conductive, stretchable, adaptable, self-healing, and biocompatible liquid metal GaInSn/Ni-based composite hydrogel is developed by incorporating a magnetic liquid metal into the hydrogel framework through crosslinking polyvinyl alcohol (PVA) with sodium tetraborate. The excellent stretchability and fast self-healing capability of the PVA/liquid metal hydrogel are derived from its abundant hydrogen binding sites and liquid metal fusion. Significantly, owing to the magnetic constituent, the PVA/liquid metal hydrogel can be guided remotely using an external magnetic field to a specific position to repair the broken wires with no need for manual operation. The composite hydrogel also exhibits sensitive deformation responses and can be used as a strain sensor to monitor various body motions. Additionally, the multifunctional hydrogel displays absorption-dominated electromagnetic interference (EMI) shielding properties. The total shielding performance of the composite hydrogel increases to ~ 62.5 dB from ~ 31.8 dB of the pure PVA hydrogel at the thickness of 3.0 mm. The proposed bioinspired multifunctional magnetic hydrogel demonstrates substantial application potential in the field of intelligent wearable devices.

**Supplementary Information:**

The online version contains supplementary material available at 10.1007/s40820-023-01043-3.

## Highlights

- A conductive, stretchable, adaptable, and self-healing, GaInSn/Ni--based composite hydrogel by incorporating a magnetic liquid metal into the hydrogel framework through crosslinking polyvinyl alcohol with sodium tetraborate.
- The multifunctional composite hydrogels showed outstanding performance for magnetic repair movement sensing, and EMI shielding.

**Supplementary Information:**

The online version contains supplementary material available at 10.1007/s40820-023-01043-3.

## Graphical abstract

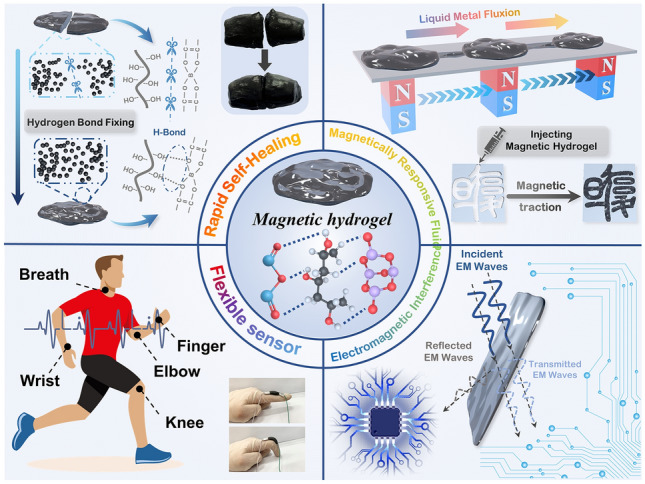

## Introduction

Liquid metals of Ga-based alloys have attracted intensive interest because of their deformability, nontoxicity, self-healing capability, high electric/thermal conductivity, and unique surface chemistry [1–3]. In particular, the fluidity and deformability of liquid metals in aqueous environments are promising for applications in various fields, such as flexible electronics [4–6], soft robotics [7, 8], energy harvesting/storage [9, 10], microfluidics [11], sensors [12], actuators [13, 14], and wearable sensing [15].

Currently, miniaturized, integrated, and high-power electronic devices are being rapidly developed for wireless communication, and significantly large electromagnetic interference (EMI) is produced as an inevitable by-product [16, 17]. EMI has a substantial effect on the nearby electronic apparatus and may result in the degradation and malfunctioning of electronics, particularly those working at high frequencies [18–20]. Furthermore, EMI pollution has severe adverse effects on the surrounding environment and human health. Therefore, the development of EMI functional materials has become a significant alternative to effectively alleviate this dilemma [21–23]. Compared with traditional rigid metals, MXenes and carbon nanomaterials [24–26], liquid metals have emerged as promising EMI shielding materials and attracted increasing attention because of their excellent processability, good fluidity, and high conductivity [27–29]. Zhu et al. fabricated a flexible multifunctional EM shielding film derived from an Ecoflex elastomer filled with magnetic liquid metal droplets [30]. The film exhibited strain-improved electrical conductivity and EMI shielding properties when subjected to uniaxial tensile stress. A three-dimensional (3D) liquid metal network was inserted in a stretchable polydimethylsiloxane composite foam, and the resulting material demonstrated significant EMI shielding enhancement under compression and stretching [31, 32]. The 3D liquid metal configuration could regulate the electrical conductivity during stretching and compression, enhancing the reflection of EM waves and shielding the EMI. Liquid metal-based monoliths with a 3D continuous conductive network were successfully prepared using a constrained thermal expansion method [33]. The as-prepared monoliths possessed tunable architectures owing to the microcosmic fluidity of the liquid metal and demonstrated excellent EMI shielding performance. Huang et al. developed a self-standing thermostable composite film for EMI shielding by introducing a small quantity of aramid nanofibers into liquid metals [34].

However, the EMI shielding properties of liquid metal-based composites are mainly achieved by reflecting electromagnetic waves, which will cause secondary pollution. Moreover, their limited self-healing ability hampers their applications in intelligent, flexible devices. In contrast to metal liquid-filled elastomers, hydrogels are a type of engineering material composed of a crosslinked network of hydrophilic blocks surrounded by water [35], demonstrating the potential for absorption-based EMI blocking [36]. Moreover, compared to their dry counterparts, soft polymer hydrogels possess shape adaptability and self-healing capabilities, enabling stable and conformal interfaces with blocked targets for applications in artificial skin or wearable electronics [37–39]. In the past decades, fast self-healing, shape/size-tunability, and EMI shielding properties of hydrogels have been extensively studied. Accordingly, several hydrogels have been developed, such as sandwich-structured hydrogels consisting of a layer of silver nanowire and two layers of polyvinyl alcohol (PVA) hydrogels reinforced by aramid nanofibers [40], Ti_3_C_2_-MXene-functionalized poly(3,4-ethylenedioxythiophene):polystyrene sulfonate (PEDOT:PSS) hydrogels [41], ionic liquid doped PEDOT:PSS hydrogels [42], polyacrylic acid/cellulose nanofibers/MXene/calcium ion composite hydrogel [43], MXene organohydrogel containing glycerol and water binary solvents [44], and multiwall carbon nanotubes/polyacrylamide/cellulose nanofiber hydrogels [45]. However, these hydrogels repair themselves by the aid of external manual operation. The fabrication of self-healing liquid metal-based soft hydrogels with no need for manual touch remains an open challenge.

Herein, we present a simple ultrasonic method to fabricate PVA/GaInSn–Ni composite hydrogels with rapid self-healing capability and excellent stretchability and shape adaptability. The multifunctional composite hydrogels demonstrated high performance for magnetic repair and prototyping, body movement sensing, and EMI shielding. The abundant reversible hydrogen bonds between PVA and borate ions and the fluidity of liquid metals render the PVA/liquid metal hydrogel self-healing features in the absence of any external stimulus. In the presence of a magnetic field, broken wires can be repaired remotely by placing them in a sealed space with one end warped by the magnetic liquid metal hydrogel. The composite hydrogel could be used as a strain sensor to detect body motions and as a signature sensor that sensitively and rapidly responds to various external stimuli. The water-rich hydrogel with moderate conductivity provides absorption-dominated EMI shielding performance to the composite. Importantly, the composite hydrogel exhibited long-term stability for EMI shielding even after storage for 1 year.

## Experimental

### Raw Materials

PVA (Mw ≈ 145,000) was purchased from Meryer Co., Ltd. Sodium tetraborate (anhydrous, 99%), gallium (99.9%), indium (99.9%), and tin (Sn, 99%) were obtained from Shanghai Macklin Biochemical Co., Ltd. Nickel microparticles (99.9%) were obtained from Shengshida Metal Materials. Co. Ltd., China. All reagents were used as received without further modification.

### Synthesis of EGaInSn Droplets

The EGaInSn liquid metal alloy was fabricated by melting a mixture of Ga (68 wt%), In (22 wt%), and Sn (10 wt%) in an oil-bath pan at 150 °C for 1 h.

### Preparation of PVA/EGaInSn–Ni Composite Hydrogels

First, PVA white powder (0.4 g) was added to 5 mL of deionized (DI) water and magnetically stirred at 80 °C for 7 h until all powders were dissolved to obtain a PVA solution (8 wt%). Thereafter, EGaInSn and Ni were added to the solutions, and then, EGaInSn–Ni was evenly dispersed in the solution by ultrasonic treatment for 2 h. Meanwhile, sodium tetraborate was dissolved in hot DI water (60 °C) and shaken well until the particles were completely dissolved to produce a solution of 4 wt%. After cooling to room temperature, the sodium tetraborate solution was slowly blended with the PVA mixture to prepare composite hydrogels. PVA-based hydrogels with different mass ratios of EGaInSn and Ni, namely 1:0.5, 1:1, 1:2, 1:4, and 1:8, are denoted as PVA/EGaInSn–0.5Ni, PVA/EGaInSn–1Ni, PVA/EGaInSn–2Ni, PVA/EGaInSn–4Ni, and PVA/EGaInSn–8Ni, respectively.

### Characterization

A powder X-ray diffractometer (XRD, Bruker, D8 ADVANCE) was used to record the XRD patterns. X-ray photoelectron spectroscopy (XPS) analyses were performed on a Kratos AXIS Ultra spectrometer equipped with a monochromatized Al Kα X-ray source. Fourier transform infrared (FTIR) spectra were acquired using a spectrometer (Bruker, Vertex80V). The functional groups of the PVA-based hydrogels were examined using an FTIR spectrometer (Thermo Nicolet iS10). The morphology and elemental distribution of the PVA–LM hydrogels were characterized using Scanning electron microscopy (SEM, Nova Nano SEM 450) coupled with energy-dispersive X-ray spectroscopy (EDS, Bruker Silicon). The rheological behavior of the hydrogels was investigated using a Thermo HAAKE MARS 60 machine with a 20 mm parallel plate. A dynamic frequency sweep of 0.1–10 Hz was conducted at 25 °C with a fixed oscillation strain of 0.2% in the oscillation mode. The electrical resistivities of the PVA/EGaInSn–Ni and pure PVA hydrogel samples were tested using an RTS-8 four-probe resistivity meter. An LCR instrument (TH2830) operated using a LabView software was used to collect all relevant data. In the EMI shielding measurement, the composite hydrogel is cut into rectangle shape with dimensions of 22.86 mm × 10.16 mm × 3.0 mm. The EMI shielding properties of the PVA/liquid metal composite hydrogels were measured using a vector network analyzer (N5234B, KEYSIGHT) in the X band (8.2–12.4 GHz), and more details can be found in the Supporting Information.

## Results and Discussion

### Characterization of PVA–Liquid Metals Hydrogels

**Fig. 1:**
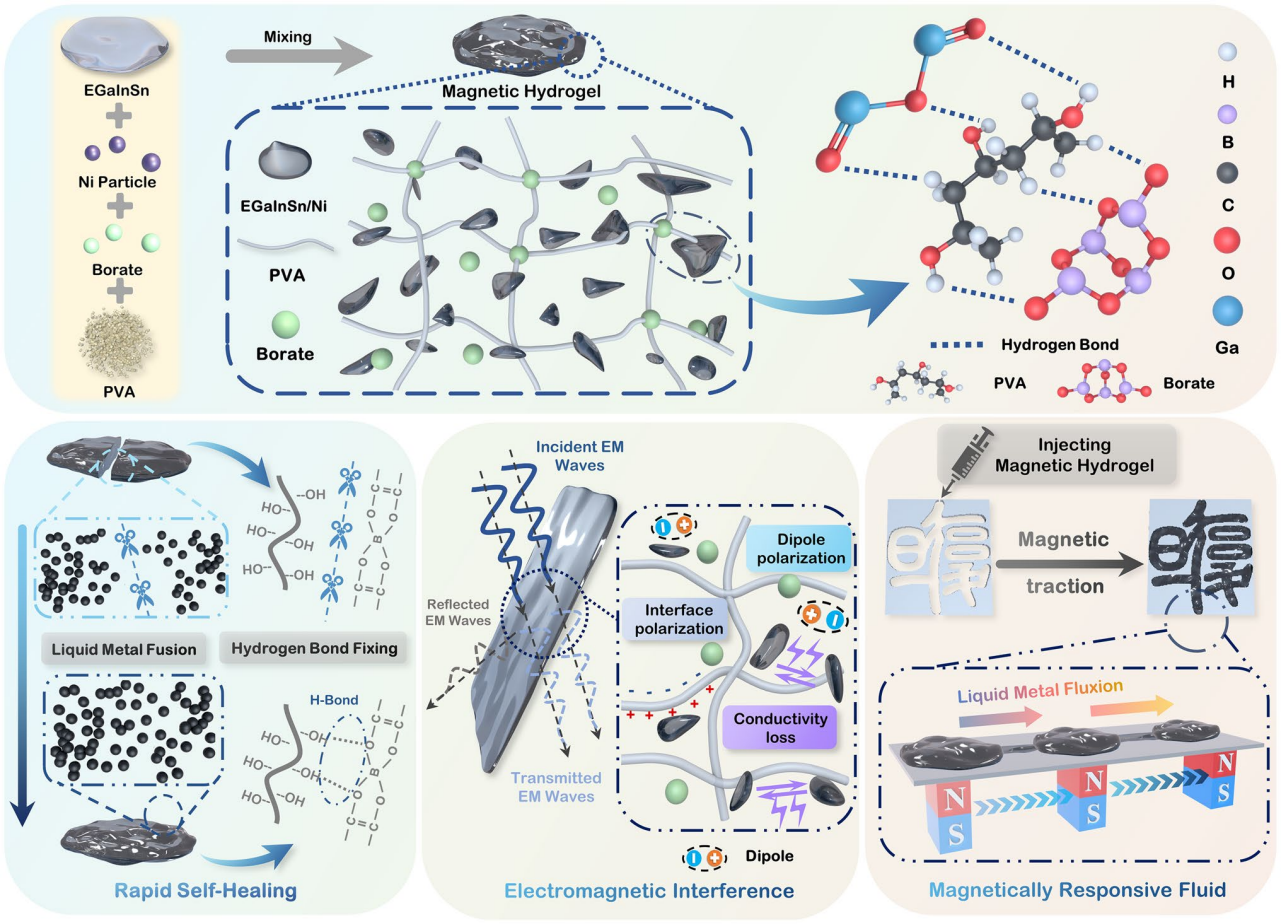
Fabrication of the self-healing liquid metal hydrogel. **a** Scheme illustrating the preparation of the PVA/EGaInInSn–Ni composite hydrogel containing primary crosslinked networks. **b** Self-healing mechanism of the PVA/EGaInInSn–Ni hydrogel. **c** Schematic demonstrating the high-performance EMI shielding of the liquid metal-based hydrogel resulting from conductive loss, interfacial polarization, and dipole polarization. **d** Schematic showing external magnetic force can move composite hydrogel

Figure 1a illustrates the fabrication process of the self-healing PVA/EGaInSn–Ni composite hydrogel. A eutectic GaInSn (EGaInSn) suspension was first prepared by the ultrasonic treatment of Ga, In, and Sn metals with a specific mass ratio. The composite PVA/EGaInSn–Ni was obtained by mixing the EGaInSn suspension with Ni particles, PVA, and sodium tetraborate. Herein, borate molecules acted as crosslinkers by forming abundant hydrogen bonds with PVA [46, 47]. Previous studies showed that a thin oxide skin layer could be easily formed on the surface of gallium-based liquid metals in the air [48], leading to secondary crosslinking with PVA chains. The as-fabricated PVA/EGaInSn–Ni hydrogel exhibits excellent self-healing capability (Fig. 1b), which is derived from the abundant H-bond sites. The self-healing properties of the prepared hydrogels are mainly attributed to two factors: (1) As a self-healing supramolecular adhesive, PVA polymer not only has certain viscosity, but also has complex network structure and good biocompatibility, which can promote fluidity, ductility and self-healing. Polymerization of PVA with borax by diol results in many network structures, in which the hydroxyl group in the PVA chain forms a weak hydrogen bond with the borax molecule, forming a “tenon-like” structure. After the material is broken, the exposed new surface is rich in PVA hydroxyl group and borax molecule. When the two damaged surfaces contact, the weak hydrogen bonds formed between the hydroxyl group in the PVA chain and borax molecule gradually recombine and connect, which leads to the material healing. (2) Hydrogen bonds between the hydroxyl group of the PVA chain and the thin metal oxide layer on the liquid metal surface provide abundant sites for molecular crosslinking during self-healing, which can be repaired quickly after interconnection. Additionally, the fluidity of liquid metal further facilitates the crosslinking of the PVA hydrogel and promotes self-healing. These PVA–liquid metal composite hydrogels are expected to have excellent EMI shielding properties (Fig. 1c) because of the conductive networks of liquid metals, abundant dipoles in water, and numerous interfaces between PVA, EGaInSn, and Ni. Adding magnetic Ni particles allows the composite hydrogels to move under a magnetic force (Fig. 1d).

The EGaInSn particles were synthesized through an isothermal sonication process at 150 ℃ for 60 min. The long sonication process yields highly dispersed liquid metal droplets with uniform elemental distribution (Fig. S1). A more detailed study on the surface composition of EGaInSn was performed using XPS (Fig. S2). In the XPS spectra of Ga 3*d* (Fig. S2a), the peak at 18.5 eV is associated with metallic gallium (Ga^0^) [49], while two peaks at 19.6 and 20.6 eV correspond to the chemical states of Ga^1+^ in Ga_2_O and Ga^3+^ in Ga_2_O_3_ [50], respectively. The O 2*s* peak at 23.8 eV is also observed in the Ga 3*d* spectrum, confirming the oxidation of Ga [51]. The functional shell on the liquid metal droplets had numerous oxygen-containing groups, which were expected to significantly enhance the interaction between the droplets and polymer matrix. In the In 3*d* core-level spectrum, two distinct peaks at 443.1 (In 3*d*_5/2_) and 450.7 eV (In 3*d*_3/2_) are attributed to In^0^ (Fig. S2b). Similarly, in the Sn 3*d* XPS spectrum (Fig. S2c), the peaks centered at 484.8 and 493.2 eV are related to the metallic Sn^0^. These results suggest that the surface of the EGaInSn particles a mainly composed of Ga oxides and a small amount of the mixture of In and Sn oxides. The O 1*s* XPS spectrum (Fig. S2d) is deconvoluted into two peaks at 531.7 and 533.3 eV, suggesting the presence of intact stoichiometric oxides and oxygen vacancies, respectively [52].

**Fig. 2:**
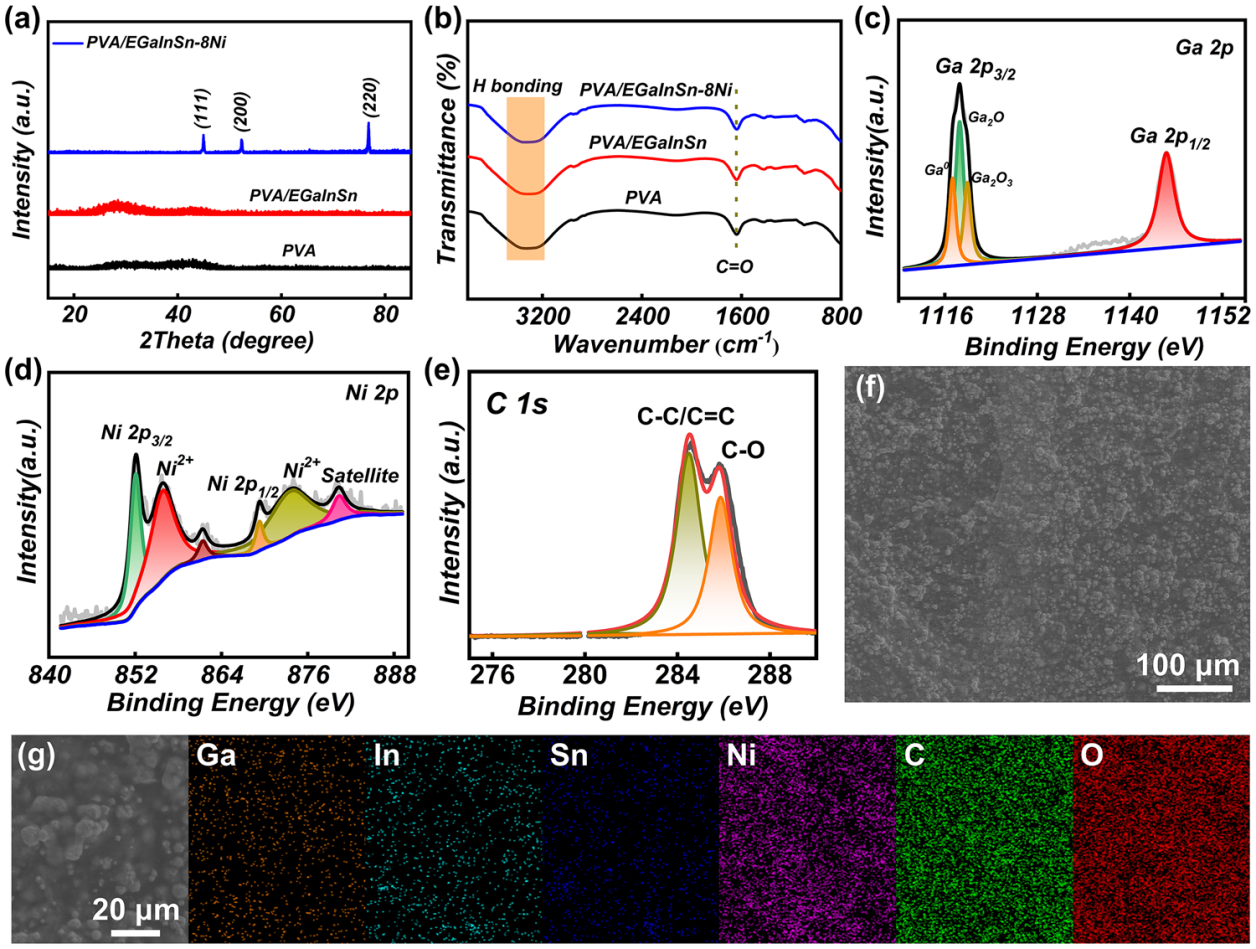
Material characterizations of PVA/EGaInInSn–Ni composite hydrogels. **a** XRD patterns and **b** FTIR spectra of pure PVA, PVA/EgaInSn, and PVA/EGaInInSn–8Ni hydrogels. High-resolution XPS spectra of **c** Ga 2*p*, **d** Ni 2*p*, and **e** C 1*s* in the PVA/EGaInInSn–8Ni hydrogel. **f** SEM images of PVA/EGaInInSn–8Ni and **g** corresponding element mapping of Ga, In, Sn, Ni, C, and O

Figure 2a displays the powder XRD patterns of pure PVA, PVA/EGaInSn, and PVA/EGaInSn containing 8 wt% Ni particle (PVA/EGaInSn–8Ni) hydrogels. No distinct peaks are observed in the PVA hydrogel, confirming its crosslinking state [53]. After PVA is mixed with EGaInSn liquid metal, a low-intensity broad peak at ~ 35° appears, indicating the presence of amorphous components (e.g., amorphous oxides and/or the liquid itself) [54]. With the introduction of Ni particles, three clear diffraction peaks at 44.6°, 52.0°, and 76.5° are observed, which are indexed to the (111), (200), and (220) planes of the face-centered cubic structure of Ni (JCPDS no. 04–0850). Similar to PVA/EGaInSn–8Ni, all XRD patterns of the hydrogels with various Ni contents show three diffraction peaks of Ni (Fig. S3). FTIR spectroscopy provides more detailed information for elucidating the crosslinked structure (Figs. 2b and S4). Pure PVA, PVA/EGaInSn, and PVA/EGaInSn–Ni hydrogels exhibit a strong and broad peak at 3400 cm^−1^ due to the absorption characteristic of water and stretching vibration of the –OH group, which is a typical indication of hydrogen bonding [55]. The peak associated with C–O stretching vibration is observed at 1630 cm^−1^. The carbonyl (C=O, the acceptors of hydrogen bonds) and hydroxyl groups (–OH, the donors of hydrogen bonds) can produce a high density of hydrogen bonds [56], which is consistent with the broad and strong peak at 3455 cm^−1^.

The chemical bonding states of the PVA/EGaInSn–8Ni composite hydrogels were confirmed using XPS (Fig. 2c–e and S5). The Ga 2*p* core-level spectrum (Fig. 2c) is deconvoluted to three peaks related to Ga^+^, Ga^3+^, and metallic Ga. The high-resolution XPS spectrum of Ni 2*p* (Fig. 2d) shows the coexistence of Ni^0^ and Ni^2+^ species [57], indicating that oxidation occurred on the surface of the Ni particles. In the In 3*d* region (Fig. S5a), the metallic indium exhibits two peaks centered at 443.7 (In 3*d*_5/2_) and 451.4 eV (In 3*d*_3/2_), with a separation of spin–orbit components $\Delta_{{{\text{metal}}}}$ ≈ 7.7 eV [58]. In the Sn 3*d* XPS spectrum (Fig. S5b), the peaks centered at 484.9 and 493.5 eV correspond to the metallic tin [59]. As shown in Fig. 2e, the C 1*s* core-level spectrum has three-peak components, i.e., C–C/C=C (284.9 eV) and C–O (286.5 eV). The strong C signal indicates the presence of abundant hydrophilic groups on the PVA chains, which offer abundant crosslinking sites in the hydrogel [36]. The O 1*s* peaks are deconvoluted to metal oxide, hydroxide, and lattice oxide component peaks (Fig. S5c), demonstrating the formation of mixed liquid metal oxides and the Ni oxidation states on the shell [60]. SEM images illustrate the uniform dispersion of liquid metal particles containing Ni in the PVA polymer (Fig. 2f, g). Corresponding elemental mapping using EDS reveals that Ga, In, Sn, and Ni elements are confined in the particles, while the C and O elements are observed in all the SEM images, which is in good accordance with the hydrogel composition. In these PVA–liquid metal composite hydrogels, the water contents are high, with slight differences among the PVA hydrogels with introduction of liquid metals. As shown in Fig. S6, the water contents of PVA hydrogels decreased with increase in liquid metal amounts, and the water content of the original PVA-EGaInSn–8Ni hydrogel sample can reach as high as about 74 wt%.

### Self-healing, Magnetic-Drag and Smart Responsible Properties of Hydrogels

The rheological properties of the liquid metal hydrogels were studied using oscillatory rheology (Fig. S6). For pure PVA and PVA/EgaInSn hydrogels, their loss modulus (*G*″) values are smaller than the storage modulus (*G*) in the frequency range of 1–10 Hz, indicating their solid-like states. Significantly, the *G*″ value of PVA/EGaInSn–8Ni exceeds *G′*, exhibiting a liquid-like state (Fig. S7a). Therefore, Ni in the hydrogels facilitated the generation of a more liquid-like condition without damaging the complex network structure. Furthermore, for the liquid metal hydrogels with different Ni contents (Fig. S7b), the *Gʹ* of all composite hydrogels is less than *G*″, confirming their liquid-like behaviors. Therefore, for the subsequent experiments, we chose PVA/EGaInSn–8Ni as the smart sensor hydrogel.

**Fig. 3:**
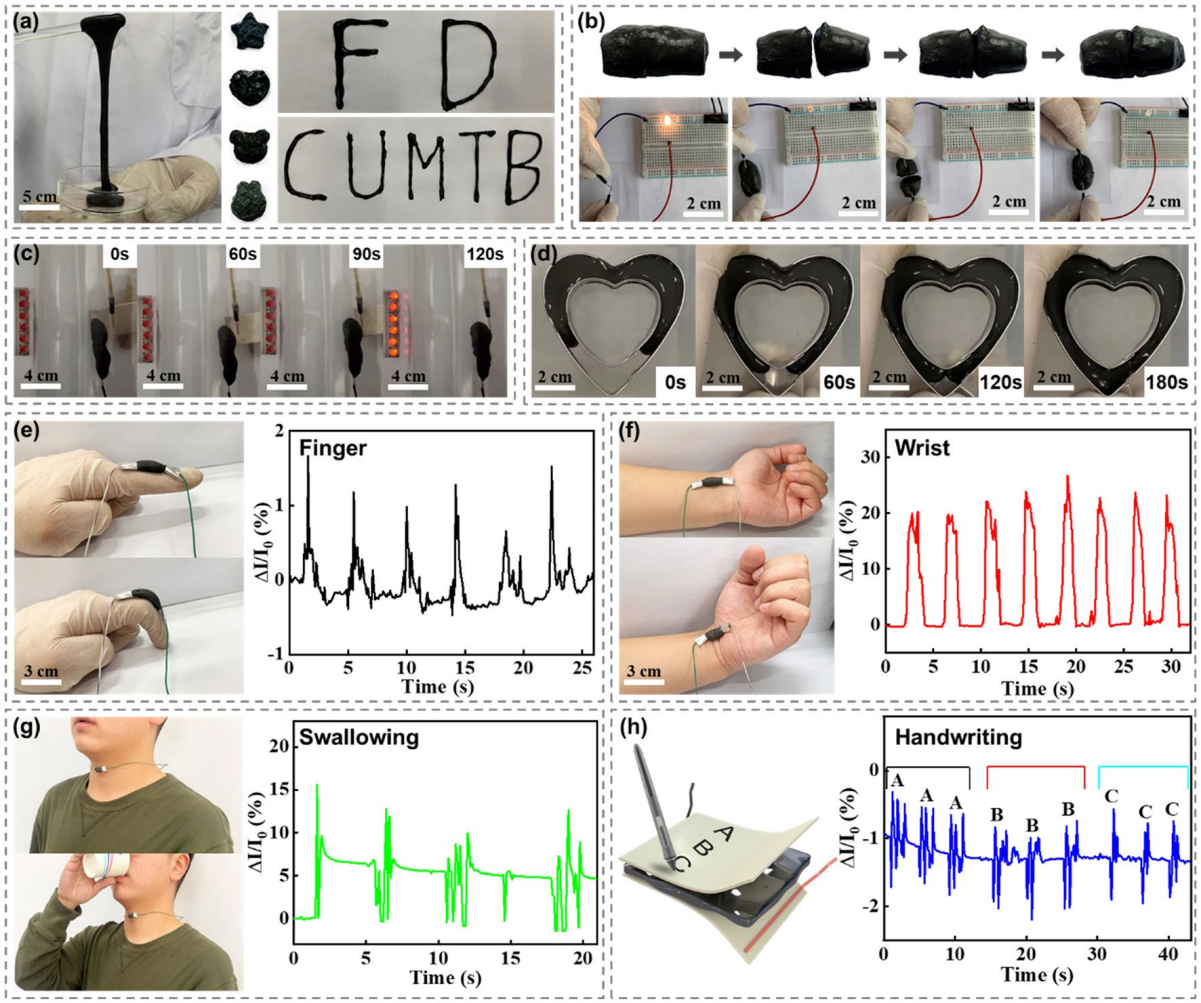
Flexible, self-healing, magnetic-responsive properties of the liquid metal composite hydrogel. Optical photographs demonstrating **a** stretchability, plasticity, and writability; **b** automatically self-healing capability; **c** magnetic field-driven movement for the junction circuit; **d** magnetic field-induced prototyping. Relative current changes of the liquid metal hydrogel used as a pressure sensor for the real-time detection of **e** finger bending, **f** wrist bending, and **g** swallowing. **h** PVA/EGaInInSn–8Ni hydrogel sandwiched between two layers of polyimide films to real-time monitor the handwriting of English letters “A/B/C”

The black adhesive PVA/EGaInSn–8Ni hydrogel is in a chuddy‐like state with multifunctional performance. Its stretchability easily reaches more than 800% (Fig. 3a). The liquid metal hydrogel also demonstrated excellent plasticity and writability. It could adapt to the desired shape, and the formed shape could be broken and pasted onto various objects in different shapes, such as starlike, heart, face-like shapes, indicating the shape-controllable feature of composite hydrogels. During the deformation process, the hydrogel remained intact without cracks. Owing to its good liquid mobility, the hydrogel inks were used to write any words, including “FD” and “CUMTB.” The as-fabricated PVA/EgaInSn–8Ni also has significant self-healing capability (Fig. 3b). When two separate parts touched each other in the natural environment, they joined seamlessly and rapidly. The high self-healing performances were derived from the intensive hydrogen bonding and liquid metal fusion that facilitated PVA crosslinking. Moreover, an emitting diode was used to illustrate the self-healing performance of the liquid metal composite hydrogel. When the power supply, wires, diode, and hydrogel were connected to form a loop, the diode was lit up. When the hydrogel was broken, the diode was turned off. Once the broken hydrogel was joined, the diode glowed again. Figure 3c illustrates a typical hydrogel repair of wire joints guided by an external magnetic field because the PVA/EGaInSn–8Ni hydrogel possesses the specific saturation magnetization (*M*_s_) value of 12.2 emu g^−1^ (Fig. S8). The disconnected wire, in which one end is wrapped in the hydrogel, is placed in a plastic container. A permanent magnet is placed outside the plastic container. When the magnet is moved closer to the hydrogel, the entire hydrogel shifts toward the magnet. The strong adhesion between the liquid metal and PVA prevents the liquid metal droplets from leaving the hydrogels during magnetic field-driven movement. The magnet can drag the hydrogel to repair the wire conduction and finally lit the diodes up (Movie S1). In addition, the liquid metal hydrogel can be guided using the magnetic field to fill the custom-designed heart-shaped pattern (Fig. 3d and Movie S2).

The high stretchability, plasticity, writability, self-healing, and magnetic field-induced repair and shaping properties enable the application of PVA/liquid metal hydrogels as strain sensors for detecting various body movements. This magnetic liquid metal hydrogel was significantly soft and flexible and could easily adhere to different joints of the human body. The PVA/liquid metal hydrogel was attached to the index finger to monitor finger flexion, as shown in Fig. 3e. The uniform sensing signal and distinct signal change ($\Delta I/I0$, $\Delta I$: change current under strain stimuli; *I*0*:* original current) indicate the good reliability of the magnetic PVA/liquid metal hydrogel monitoring. When the PVA/liquid metal hydrogel is attached to the wrist, the current increases when bending the wrist, and the current–time curve exhibits significant stability and reproducibility (Fig. 3f). Therefore, the PVA/liquid metal hydrogel sensor demonstrates considerable potential for real-time human health monitoring [61]. Based on the compression effect generated by vocal cord vibration [62], when the hydrogel sensor is attached to the throat, swallowing can also be clearly identified (Fig. 3g). The accurate detection of swallowing during water drinking suggests that the sensor can be used to create a pharyngeal motion recognition device. Additionally, the hydrogel sensor could accurately capture the frequency of the laryngeal vibrations. Moreover, when the hydrogel sensor was coated with two layers of polyimide film on its surface, it acted as a smart-writing keyboard to sense the signature on its surface. When the letters “A,” “B,” and “C” are written on the hydrogel sensor (Fig. 3h), various forms of signals are generated. These results showed that magnetic PVA/liquid metal hydrogels have significant application potential in flexible wearable electronics.

### EMI Shielding Properties of PVA–Liquid Metals Hydrogels

Electrical conductivity is crucial in determining the EMI shielding properties [63]. Therefore, the electrical conductivities of the PVA/liquid metal composite hydrogels were measured using a four-probe instrument. The electrical conductivity of the PVA/liquid metal composite is related to the migration of free ions and free water in the PVA network and the transport of electrons in the crosslinks containing EGaInSn–Ni particles (Fig. S9). As the Ni:EGaInSn ratio increased from 0.5:1 to 8:1, the conductivity of the PVA-based hydrogel gradually improved from 0.015 to 0.041 S m^−1^. More Ni content in the hydrogel prompted electron migration and thereby increased electron conduction. However, the ion channels decreased with increasing Ni concentration, resulting in the reduction of ion conduction. Therefore, the final conductivity depended on the competition between ion and electron conduction.

**Fig. 4:**
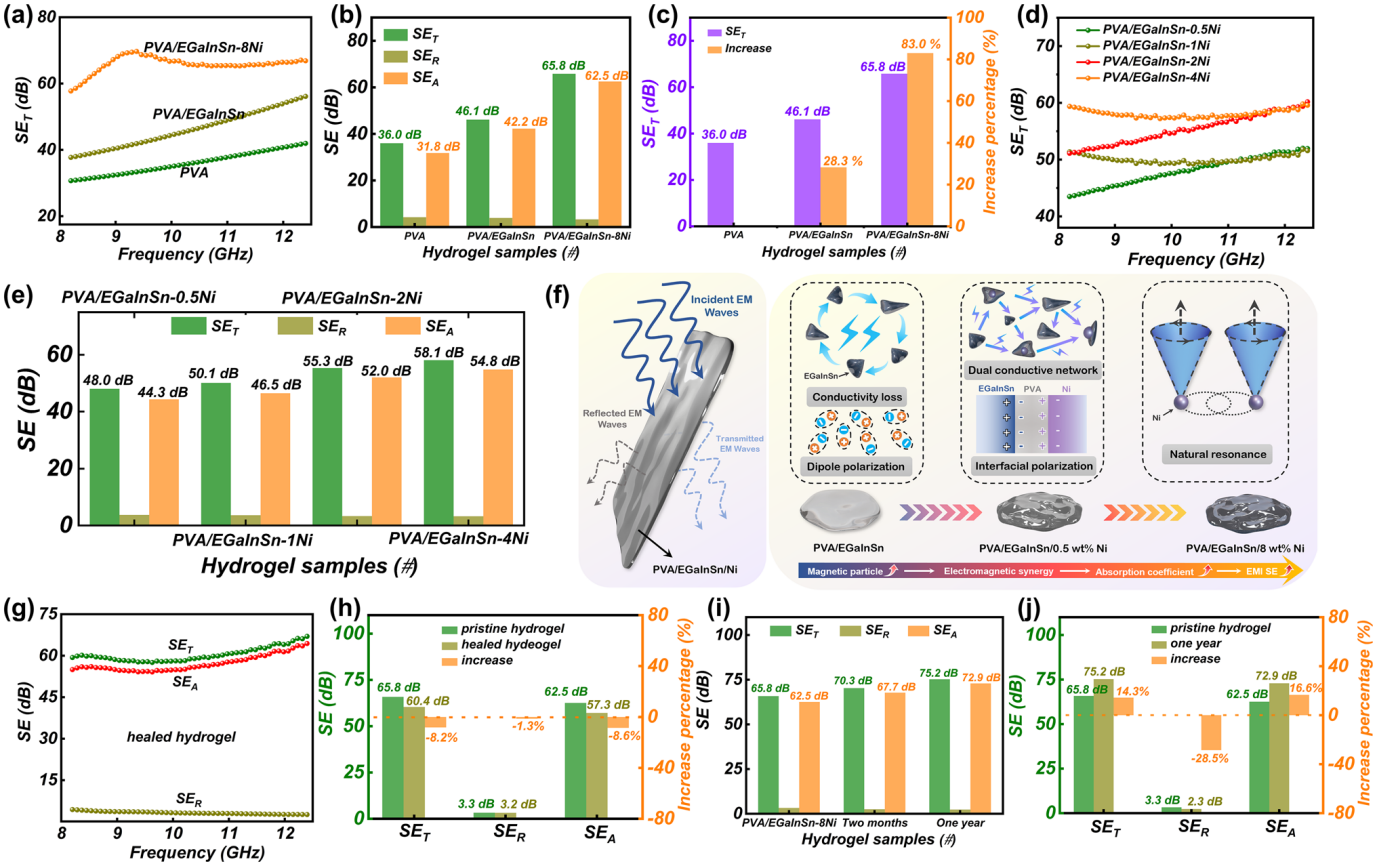
EMI shielding performances of PVA–liquid metal composite hydrogels. **a** EMI SE_T_ plots, and **b** average SE_R_, SE_A_, and SE_T_ values of PVA, PVA/EGaInSn and PVA/EGaInInSn–8Ni hydrogels at X-band. **c** EMI SE_T_ increment in PVA/EGaInSn and PVA/EGaInInSn–8Ni hydrogels compared with that of the pure PVA hydrogel. **d** EMI SE_T_ plots, and **e** average SE_R_, SE_A_, and SE_T_ values of PVA/EGaInInSn–Ni hydrogels with various Ni contents. **f** Schematic of the reinforcement effect of Ni in absorption coefficients of PVA/EGaInInSn–Ni hydrogels. **g** EMI *SE* (SE_R_, SE_A_, and SE_T_) values, and **h** SE_R_, SE_A_, and SE_T_ values in the healed PVA/EGaInInSn–8Ni hydrogel. **i** SE_R_, SE_A_, and SE_T_ values of PVA/EGaInInSn–8Ni hydrogels and **j** after storage for 1 year

EMI between electronic devices often results in equipment failure. Self-healed conductive hydrogels with good EMI properties are ideal for soft robotic applications that integrate many electronic components. The EMI shielding capabilities of PVA–liquid metal composite hydrogels (1.0 mm in thickness) were calculated by testing the scattering parameters (*S*_11_, *S*_12_, *S*_21_, and *S*_22_) in the band (8.2–12.4 GHz) using a vector network analyzer. In everyday applications, an EMI shielding effectiveness (SE) of 20 dB can block approximately 99% of incident EM wave energy [64]. As shown in Fig. 4a–c, the EMI shielding performances of PVA/EGaInSn and PVA/EGaInSn–8Ni hydrogels are significantly improved compared to that of the pure PVA hydrogel. An average total SE (SE_T_) of 36 dB is obtained in PVA, which shields 99.975% of EM waves (Fig. 4c). The SE_T_ values significantly increase to 46.1 dB (blocking 99.9975% EM waves) and 65.8 dB (blocking 99.9999747% EM waves) for PVA/EGaInSn and PVA/EGaInSn–8Ni hydrogels, respectively; the increase percentage is 28.3% and 83.0%, respectively. These results indicate that introducing liquid metals into the PVA hydrogel can prompt EMI shielding. In comparison with EMI shielding properties and sensor ability of state-of-the-art hydrogels (Table S1), our PVA–liquid metal composite hydrogels demonstrate the competitive performance for EMI shielding, magnetic repair and prototyping. The contributions from absorption loss (SE_A_) and reflection loss (SE_R_) were analyzed to assess the EMI shielding mechanism of the hydrogels (Figs. 4b, S10 and S11). The average SE_A_ values of PVA, PVA/EGaInSn, and PVA/EGaInSn–8Ni hydrogels are 31.8, 42.2 (increase percentage of 32.9%), and 62.5 dB (increase percentage of 96.8%), respectively, while the SE_R_ values decrease from 4.2 to 3.9 (decrease percentage of 7.1%) and 3.3 dB (decrease percentage of 22.0%), respectively. This demonstrates that adding liquid metals can effectively increase the absorption and decrease the reflection, and it seems that the EMI shielding mechanism mainly results from absorption. However, the calculated SE_R_ was based on the total power of EM waves, whereas SE_A_ was based on the power of incident waves [65], as reflection occurs before absorption. To further evaluate the actual shielding mechanism of the hydrogels, the power coefficients of absorption (*A*) and reflection (*R*) for the PVA, PVA/EGaInSn, and PVA/EGaInSn–8Ni composite hydrogels are shown in Fig. S12. The *R* values are higher than *A* values over the entire frequency range for the PVA and PVA/EGaInSn hydrogels, suggesting that the EMI shielding is dominated by reflection. However, in the case of PVA/EGaInSn–8Ni, the *A* value is higher than the *R* value in one-third of the X-band, indicating that introducing magnetic Ni is favorable for absorption. Although reflection dominates the EMI shielding mechanism, absorption plays an important role in the shielding contribution.

The EMI shielding properties of the PVA–liquid metal hydrogels with various Ni contents were investigated, and the results are shown in Fig. 4d. As expected, the total EMI shielding performance of the PVA–liquid metal hydrogels increases with the Ni content. To further understand the EMI shielding mechanism, the ratio of SE_R_ and SE_A_ to the overall SE_T_ were calculated, and the results are shown in Figs. 4e and S13. Distinctly, a high Ni content in PVA–liquid metal hydrogels leads to an increase in SE_A_ and a decrease in SE_R_, and the SE_A_ values are significantly higher than the SE_R_ values. However, as previously discussed, the shielding mechanism cannot be well understood based on the SE_R_ and SE_A_ values. In addition to the SE_R_ and SE_A_ values, *A* and *R* values were calculated to provide insights into the electromagnetic response [66, 67]. As shown in Fig. S14, the power coefficient *A* increases with the Ni content, whereas the *R* values exhibit an opposite trend, indicating the reinforcement effect of Ni in the absorption. In general, reflection loss is depended on the unbalanced impedance at the interfaces between the air and shields, and the absorption loss is related to the EM energy converted from the generated current and polarization relaxation [18, 68]. Figure 4f shows a schematic of the enhanced absorption coefficients resulting from the increase in the Ni content. For the PVA–liquid metal composite hydrogel, conductive networks due to free ions and liquid metals dominate the ohmic loss [25], and the abundant dipoles in PVA and free water cause strong EM loss [69]. After introducing Ni particles, numerous interfaces between water, PVA, EGaInSn, and Ni particles are created; as a result, interfacial polarization significantly dissipates EM energy [70]. Importantly, adding Ni causes magnetic loss via the main natural resonance, significantly attenuating the EM energy [71, 72]. In other words, the synergetic effects of conductive loss, dielectric loss, and magnetic loss are responsible for the enhanced EM absorption.

The EMI shielding performance of PVA/EGaInSn–8Ni before and after self-healing was verified (Fig. 4g, h). All SE_T_, SE_R_, and SE_A_ values demonstrate a slight decrease owing to the minor damage to the conductive transport networks during the cutting process. Considering the power coefficients of *A* and *R* (Fig. S15), the* R* is higher than *A* in the frequency range of 8.2–10.7 GHz; however, the* A* is higher than *R* in the frequency range of 10.7–12.4 GHz. This illustrates that the reflection dominates in most measured frequency ranges. Most conventional EMI shielding materials show deteriorated shielding performance when placed in an air atmosphere for a long time [22, 41]. Noticeably, compared with the freshly prepared hydrogel sample (about 74 wt%), the water content still maintains about 65.47 wt% after storage of 1 year (Fig. S16). Furthermore, the as-developed PVA–liquid metal composite hydrogel maintains a high EMI shielding performance (Figs. 4i–j, S17, and S18). Significantly, when stored in air atmosphere for one year, *SE*_T_ values increased from 65.8 to 75.2 dB, and SE_A_ values increased from 62.5 to 72.9 dB. The increase percentage of SE_T_ and SE_A_ reached 14.3% and 16.6%, respectively. However, SE_R_ values decreased from 3.3 to 2.3 dB, and the decrease percent was 28.5%. By analyzing power coefficients of *A* and *R* (Fig. S18)*,* PVA/EGaInInSn–8Ni presents an absorption-dominated EMI shielding mechanism after storage for one year. In an air atmosphere, hydrogels could absorb environmental water molecules to generate more free ions and dipoles, which was beneficial for EM energy dissipation and enhancing SE_T_ and SE_A_ values.

## Conclusion

In summary, we developed a multifunctional PVA/liquid metal composite hydrogel with rapid self-healing ability, excellent stretchability, shape adaptability, magnetic prototyping, sensing capability, and good EMI shielding properties. The fluidity of liquid metal and reversible hydrogen bonds between PVA chains and borate ions enabled the PVA/liquid metal hydrogel to complete self-healing rapidly without external stimuli. The proposed PVA/liquid metal hydrogel could effectively repair broken wire joints when they were placed in a precision-sealed space under an applied magnetic field. The synergy between the moderate conductivity and inherent moisture-rich environment endowed the composite hydrogel with high-efficiency EMI shielding. The total *SE* values significantly increased to 65.8 dB (blocking 99.99997% of EM waves) for PVA/EGaInInSn–8Ni hydrogels, and the increase percentage reached as high as 83.0% in comparison with those of the pure PVA hydrogel. Introducing liquid metals to PVA could effectively increase the absorption loss and decrease the reflection loss, and absorption might dominate the EMI shielding mechanism. Significantly, the excellent EMI shielding performance was maintained after storage for one year, showing long-term stability. Moreover, liquid metal hydrogels could conform to objects of arbitrary geometry and recover rapidly from damage, demonstrating significant application potential in flexible electronics and artificial skin. The hydrogel served as a strain sensor to detect various body movements and a signature sensor for sensitive and rapid responses to external stimuli. Based on the above functions, the present study offers a novel strategy to develop intelligent hydrogels for multifunctional applications as well as a versatile method for fabricating liquid metal composites with extended performance.

### Supplementary Information

Below is the link to the electronic supplementary material.

Supplementary file1 (PDF 1523 kb)

Supplementary file2 (MP4 831 kb)

Supplementary file3 (MP4 284 kb)
