# Supplementary material for: Self-Healing Liquid Metal Magnetic Hydrogels for Smart Feedback Sensors and High-Performance Electromagnetic Shielding
Source: Nanomicro Lett. 2023 Mar 31;15:79. doi: 10.1007/s40820-023-01043-3 (PMC10066054; doi:10.1007/s40820-023-01043-3)
Supplement: Supplementary file 1 — Supplementary file1 (PDF 1523 kb) [file 40820_2023_1043_MOESM1_ESM.pdf]

Supporting Information for

Self-Healing Liquid-Metal Magnetic Hydrogels for Smart Feedback Sensors and High-Performance Electromagnetic Shielding

Biao Zhao [1, 2, 5, #], Zhongyi Bai [3, #], Hualiang Lv [4, #], Zhikai Yan [5], Yiqian Du [2], Xiaoqin Guo [5], Jincang Zhang [6], Limin Wu [7], Jiushuai Deng [3], David Wei Zhang [1], Renchao Che [1, 2, 6, *]

[1] School of Microelectronics, Fudan University, Shanghai 2000433, P. R. China

[2] Laboratory of Advanced Materials, Shanghai Key Lab of Molecular Catalysis and Innovative Materials, Academy for Engineering & Technology, Fudan University, Shanghai 200438, P. R. China

[3] Key Laboratory of Separation and Processing of Symbiotic-Associated Mineral Resources in Non-ferrous Metal Industry, School of Chemical & Environmental Engineering, China University of Mining & Technology (Beijing), Beijing 100083, P. R. China

[4] Institute of Optoelectronics, Fudan University, Shanghai 200433, P. R. China

[5] Henan Key Laboratory of Aeronautical Materials and Application Technology, School of Material Science and Engineering, Zhengzhou University of Aeronautics, Zhengzhou, Henan 450046, P. R. China

[6] Zhejiang Laboratory, Hangzhou 311100, P. R. China

[7] Inner Mongolia University, Hohhot 010021, P. R. China

[#] Biao Zhao, Zhongyi Bai, and Hualiang Lv contributed equally to this work.

*Corresponding author. E-mail: rcche@fudan.edu.cn (Renchao Che)

Supplementary Figures and Tables

EMI shielding effectiveness (EMI SE), which is defined as the logarithmic ratio of incident power (P_I) to transmitted power (P_T) and described as decibels [S1], evaluates the performance of materials to shield electromagnetic waves. In general, the higher the EMI SE in dB, the less the EM wave transmits through the shields. Experimentally, the EMI SE is derived from, the scattering parameters, S_{11} and S_{21}, which are measured by a vector network analyzer (N5234B, KEYSIGHT) in the frequency range of 8.2–12.4 GHz, and their relationship is shown as [S2, S3]:

$$\text{EMI SE} = 10 \log (P_I/P_T) = -10 \log (1/|S_{21}|^2)$$

When the electromagnetic wave is incident on the surface of the shielding materials, the sum of the three power coefficients, namely, reflection (R), absorption (A), and transmission (T) must be conservative. They can be calculated by scattering parameters and expressed as:

$$R + T + A = 1$$

$$R = |S_{11}|^2 \qquad T = |S_{21}|^2$$

The total EMI SE_T is the sum of reflection (SE_R), absorption (SE_A) and multiple internal reflections (SE_M):

$$SE_T = SE_R + SE_A + SE_M$$

Generally, the multiple internal reflection (SE_M) can be neglected when SE_T is higher than 15 dB. Thus, the relationship can be simplified as [S4, S5]:

$$SE_T = SE_R + SE_A$$

The SE_R and SE_A can also be deduced using the following equations:

$$SE_R = 10 \log(1/(1-R))$$

$$SE_A = 10 \log((1-R)/T)$$

The EMI SE can be converted into the EMI shielding efficiency (%) to depict the shielding capability in unit of percentage, and relationship can be calculated based on the following equation:

$$\text{Shielding efficiency (\%)} = 100 \times [1-(1/10^{EMI\ SE/10})]$$

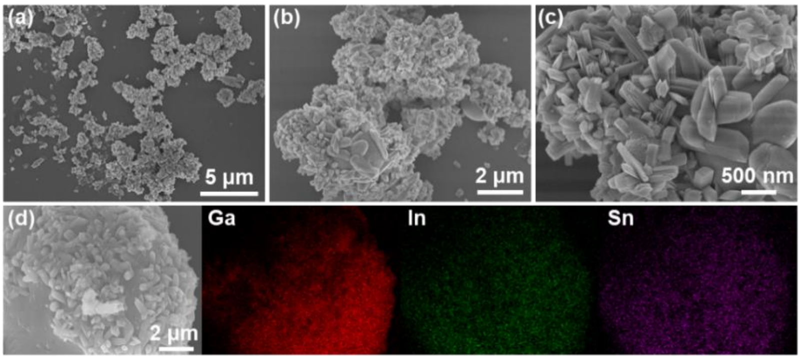

Fig. S1 (**a-c**) SEM images of EGaInSn metals. **(d)** SEM image and corresponding elemental distribution of EGaInSn sample

It can be seen from the scanning electron microscopy (SEM) images the EGaInSn droplets acquire spherical and rod-like morphologies, and the energy-dispersive X-ray spectroscopy (EDS) mappings indicates the Ga, In and Sn elements are uniformly dispersed in liquid metal droplet.

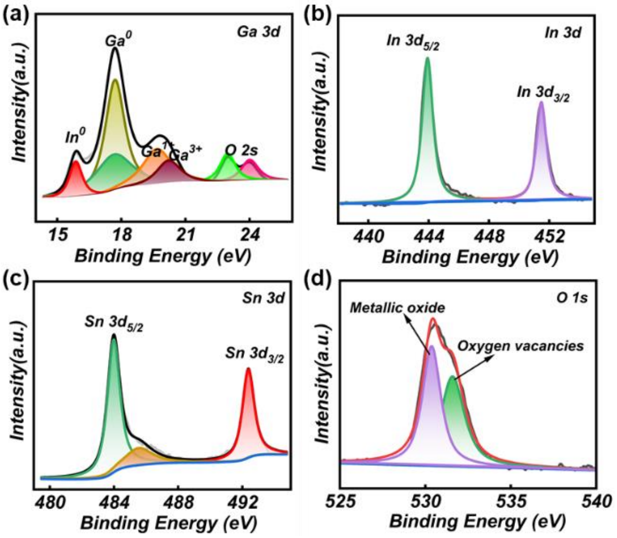

Fig. S2 High-resolution XPS spectra of (**a**) Ga 3d, (**b**) In 3d, **(c)** Sn 3d and **(d)** O 1s in the EGaInSn metal

Based on the high-resolution XPS spectra of Ga 3d, In 3d, Sn 3d and O 1, one can notice that the existence of gallium oxides can be observed in the surface of EGaInSn liquid metal.

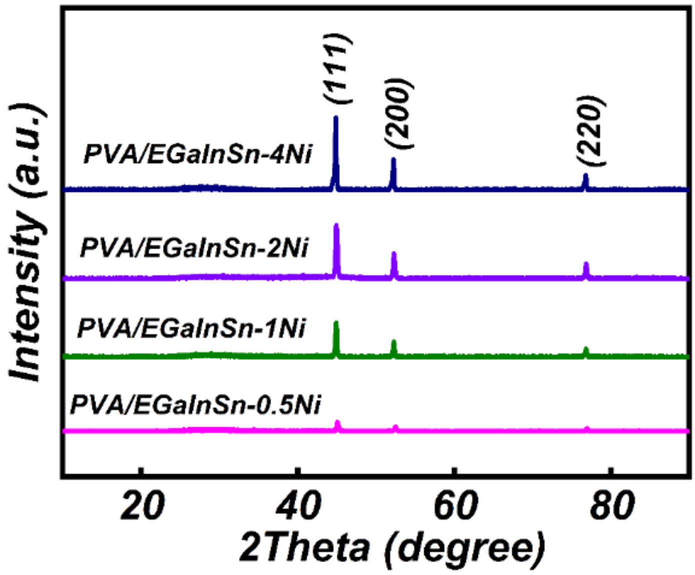

Fig. S3 XRD patterns of PVA/EGaInSn-Ni hydrogels with different Ni contents

With increasing Ni contents, the intensity of diffraction peaks attributed to Ni will be enhanced while no other diffraction peaks from additional materials are observed, which is similar with PVA/EGaInSn-8Ni sample.

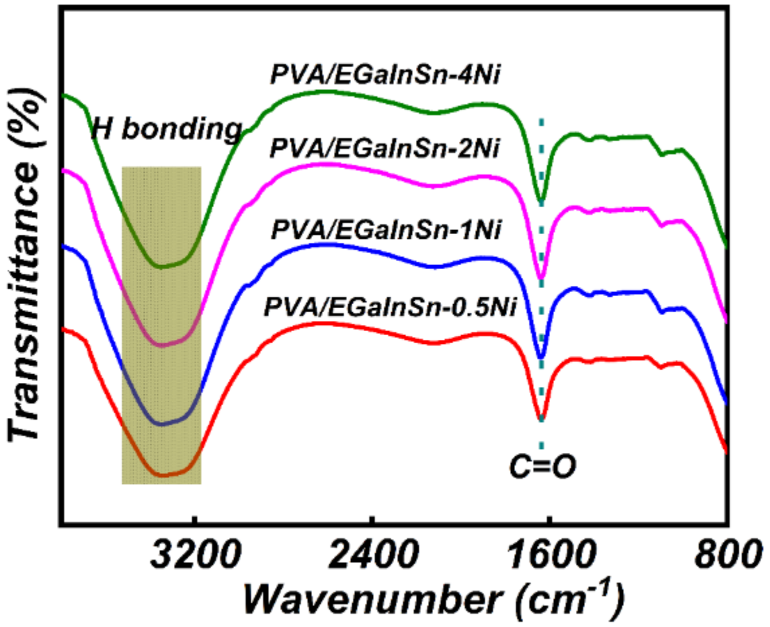

Fig. S4 FTIR spectra of PVA/EGaInSn-Ni hydrogels with different Ni contents

All PVA/EGaInSn-Ni hydrogels present similar absorption peaks in FTIR spectra, which indicate they have same functional groups. Moreover, the strong and wide spectra peaks located at 3200-3500 cm^{-1}, illustrate the formation of intense hydrogen bonds in these PVA/EGaInSn-Ni composite hydrogels.

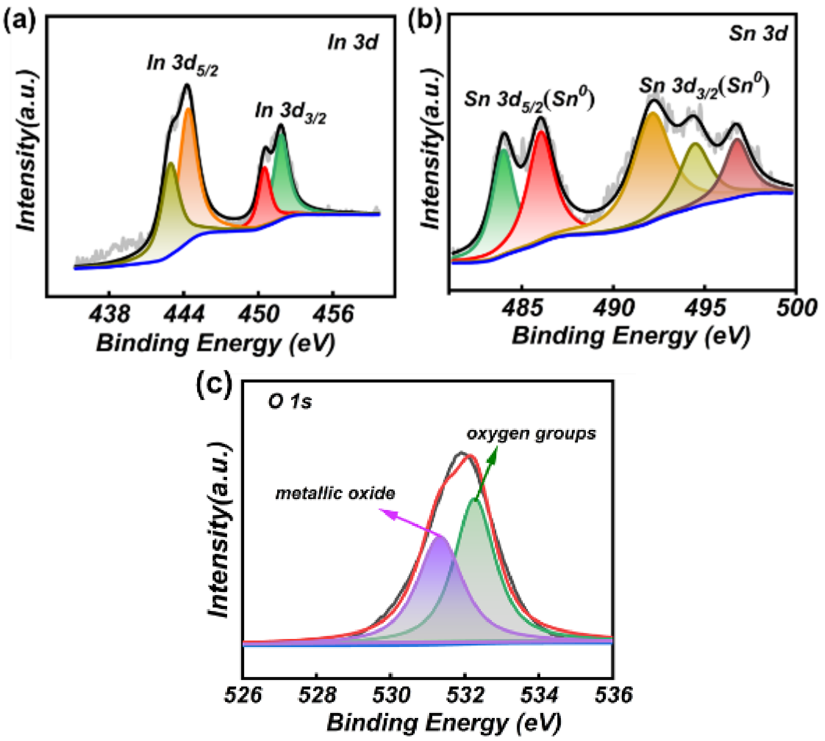

Fig. S5 High-resolution XPS spectra of (**a**) In 3d, (**b**) Sn 3d and O 1s in the PVA/EGaInSn-8Ni hydrogel

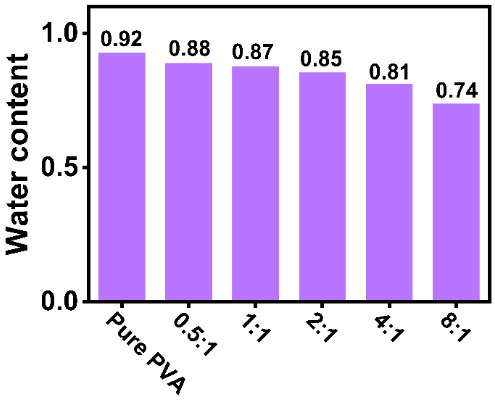

Fig. S6 The water contents of PVA-liquid metal composite hydrogels

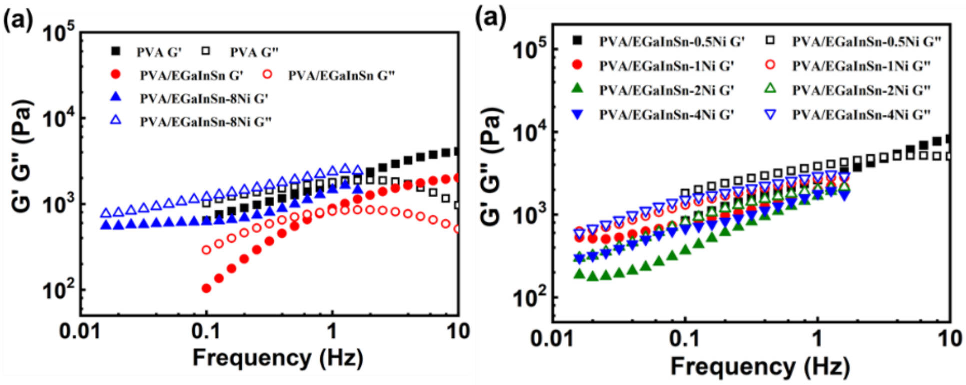

Fig. S7 Frequency dependencies of storage modulus (G′) and loss modulus (G″) for (**a**) pure PVA, PVA/EGaInSn and PVA/EGaInSn-8Ni hydrogels, and (**b**) PVA/EGaInSn-Ni hydrogels with different Ni contents

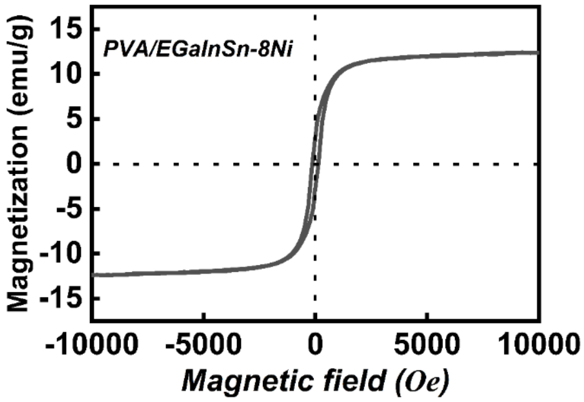

Fig. S8 The magnetic hysteresis loops of PVA/EGaInSn-8Ni composite hydrogel

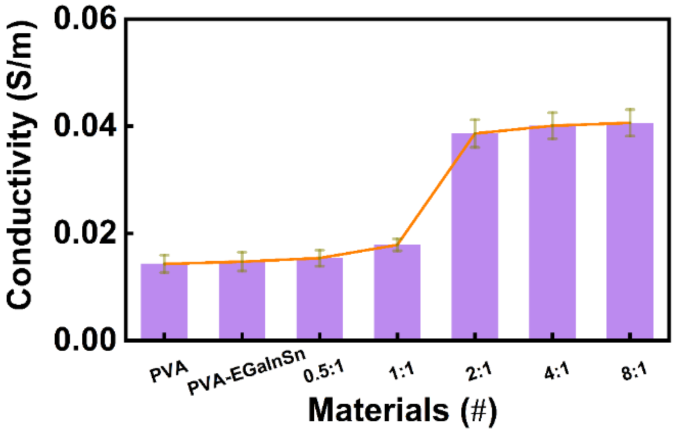

Fig. S9 Conductivity of PVA/liquid metal hydrogels with various Ni contents

Table S1 Comparison of the EMI shielding and sensor properties of recent hydrogels

Sample	SET (dB)	Thickness (t)	SE/t (dB/mm)	Effective absorbing range	Applications in the fields of smart skin, sensors and wearables	Refs.
PAM/CNF/ MWCNT hydrogels	28.5 dB	2 mm	14.25	8.2-12.4 GHz	Applied to analog skin and precision electronic protection	[S6]
polypyrrole – polyethylene glycol-PVA hydrogel	28 dB	2 mm	14	8-12 GHz	Applied to wearable field	[S7]
PNIPAAm/ PPy/cotton hydrogel	40 dB	0.37mm	108.1	8.2-12.3 GHz	/	[S8]
Nanocellulose /AgNWs hydrogel films	20 dB	52μm	384.6	8.2-12.4 GHz	Applied to analog skin	[S9]

Fe_3O_4/ PEDOT:PSS/ PVA hydrogel	46 dB	1mm	46	8-12.5 GHz	Applied to stretchable strain sensor, human-computer interaction field	[S10]
PAM/SA/ C-MXene hydrogel	26.8 dB	1mm	26.8	8.2-12.4 GHz	/	[S11]
MXene composite hydrogel	45.3 dB	0.13 mm	348.46	0.2-2.0 THz	Applied to analog skin	[S12]
Ca-PAA-CNF-MXene hydrogel	32.3 dB	2 mm	16.15	8.2-12.4 GHz	Applied to strain sensor, speech sensor, signature sensor and Morse code transmitter	[S13]
MXene hydrogel	33.6 dB	1 mm	33.6	8.2-12.4 GHz	Applied to wearable field	[S14]
PEDOT:PSS hydrogel/ EMIM-TFSI	53 dB	1 mm	53	8.2-12.4 GHz	Applied to wearable field	[S15]
MXene-functionalized PEDOT:PSS hydrogels	51.7 dB	295 μm	175.3	8.2-12.4 GHz	/	[S16]
PVA/I-P-rGO composite hydrogels	32.9 dB	1 mm	32	8.2-12.4 GHz	/	[S17]
PVA-Ni:LM hydrogels	65.8 dB	3 mm	21.9	8.2-12.4 GHz	Applied to strain sensor, speech sensor, signature sensor, magnetic repair	This work

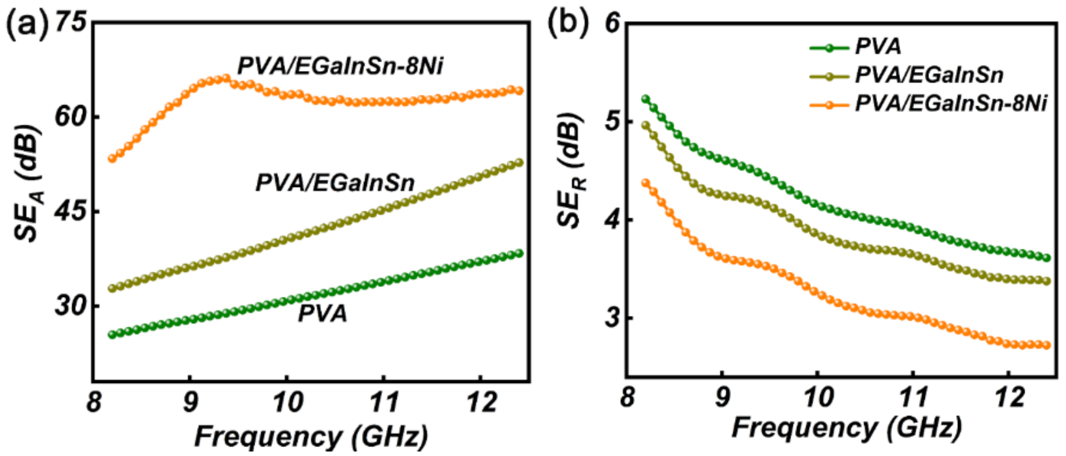

Fig. S10 (**a**) SE_A and (**b**) SE_R values of PVA, PVA/EGaInSn and PVA/EGaInSn-8Ni hydrogels at X-band

SE_A values of PVA/EGaInSn-8Ni is highest among three hydrogels while SER is the lowest in the whole X band, indicating magnetic Ni addition will beneficial for the enhancement of absorption.

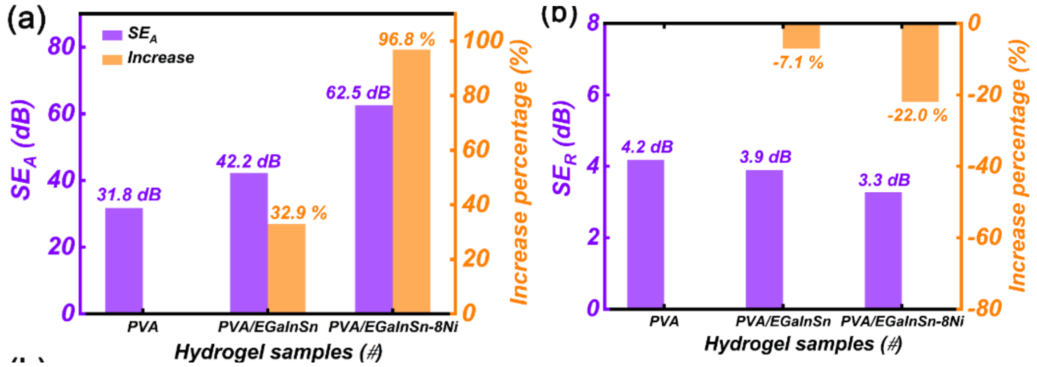

Fig. S11 (**a**) Average SE_A and increase percentage, and (**b**) SE_R values and increase percentage of PVA, PVA/EGaInSn and PVA/EGaInSn-8Ni hydrogels at X band

Based on average SE_A, SE_R values and the corresponded increase percentages (**Fig. S11**), it can be noticed that SE_A and SE_R values of PVA/EGaInSn-8Ni significantly increased and decreased, respectively, in comparison with PVA and PVA/EGaInSn hydrogels.

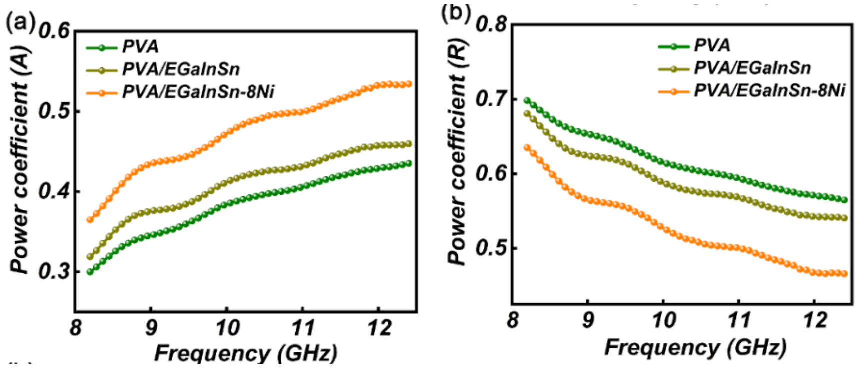

Fig. S12 (**a**) Power coefficient *A* and (**b**) power coefficient *R* of PVA, PVA/EGaInSn and PVA/EGaInSn-8Ni hydrogels at X band

The power coefficient *A* of PVA/EGaInSn-8Ni is higher than PVA and PVA/EGaInSn, specially, more than 0.5 in one third of X band, indicating absorption also makes important contribution to the shielding.

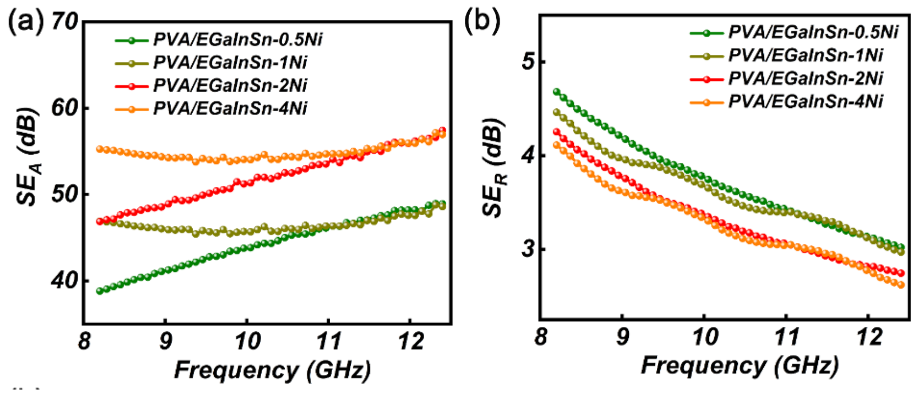

Fig. S13 (**a**) SE_A and (**b**) SE_R values of PVA-liquid metal hydrogels with various Ni contents at X band

With increasing Ni contents, note that the enhanced SE_A values of PVA-liquid metal hydrogels could be observed while the opposite tendency of SE_R values can be found, indicating Ni particles is helpful for the improvement of absorption and EMI shielding.

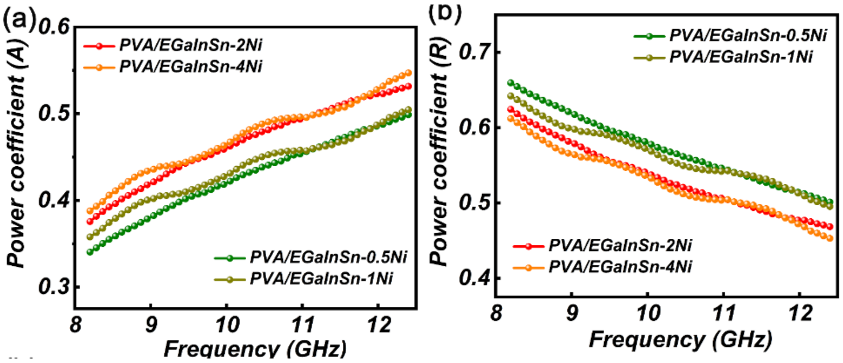

Fig. S14 (**a**) Power coefficient *A* and (**b**) power coefficient *R* of PVA-liquid metal hydrogels with various Ni contents at X band

The power coefficient *A* values of PVA-liquid metal hydrogels gradually increase with increasing Ni content, whereas power coefficient *A* values gradually decrease, indicating magnetic Ni is beneficial for the improvement of absorption.

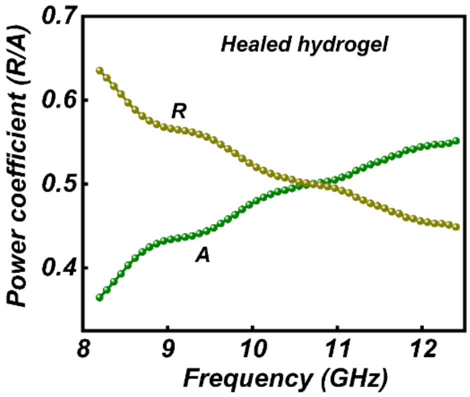

Fig. S15 Power coefficients *A* and *R* of healed PVA/EGaInSn-8Ni hydrogel

As for the healed PVA/EGaInSn-8Ni hydrogel, in the most X band, R value is higher than R, indicating reflection is dominant in the EMI shielding

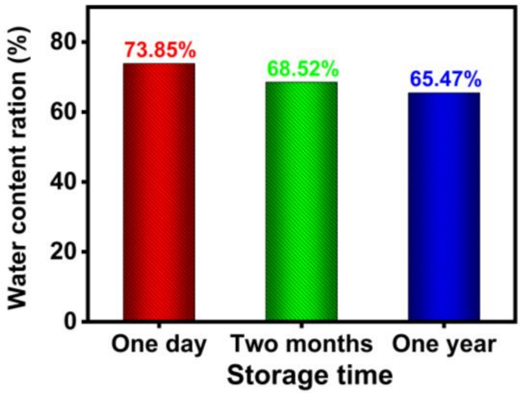

Fig. S16 Storage time-dependent water content of PVA/EGaInSn-8Ni composite hydrogel

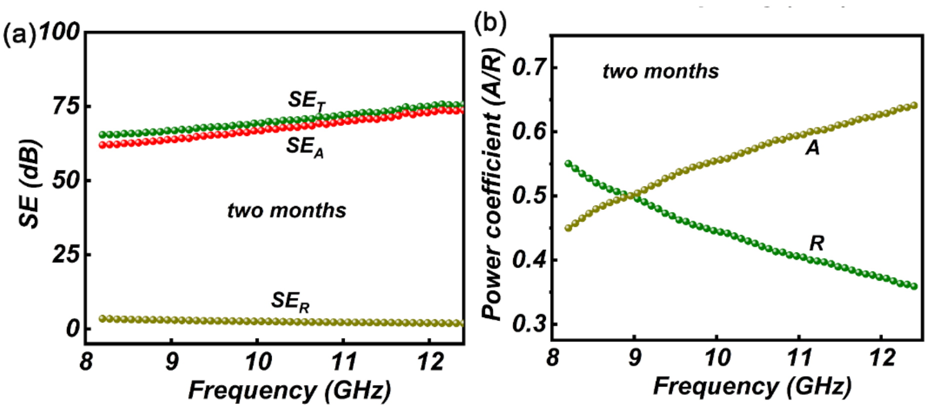

Fig. S17 (**a**) EMI shielding (SE$_T$, SE$_R$ and SE$_A$) performance and (**b**) power coefficients *A* and *R* of PVA/EGaInSn-8Ni hydrogel maintaining for two months

After placing at air atmosphere for two months, SE$_T$ and SE$_A$ display similar trend and have nearly same values. Moreover, power coefficient *A* is higher than *R* in the most X band, which suggests absorption is main EMI shielding mechanism.

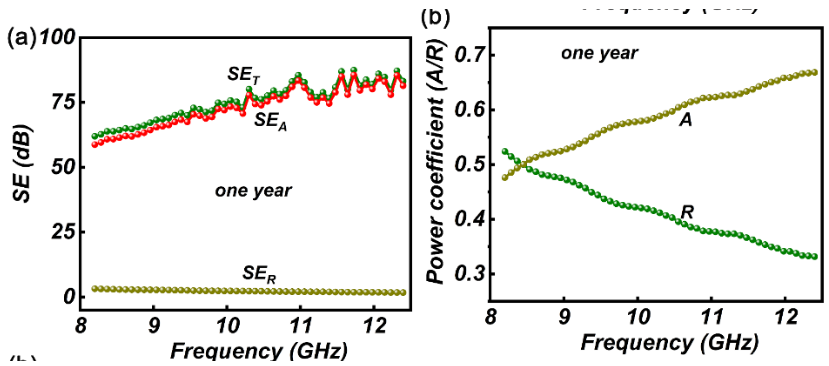

Fig. S18 (**a**) EMI shielding (SE$_T$, SE$_R$ and SE$_A$) performance and (**b**) power coefficients *A* and *R* of PVA/EGaInSn-8Ni hydrogel maintaining for one year

When placing at air atmosphere for one year, almost same values of SE$_T$ and SE$_A$ could be observed. Most important point is that power coefficient *A* is higher than *R* nearly covering the whole X band, which indicates the absorption is primary EMI shielding mechanism.

Supplementary References

[S1] Yao B, Hong W, Chen T, Han Z, Xu X et al., Highly stretchable polymer composite with strain-enhanced electromagnetic interference shielding effectiveness. Adv. Mater. **32**, 1907499 (2020). https://doi.org/10.1002/adma.201907499

[S2] Wan Y-J, Wang X-Y, Li X-M, Liao S-Y, Lin Z-Q et al., Ultrathin densified carbon nanotube film with “metal-like” conductivity, superior mechanical strength, and ultrahigh electromagnetic interference shielding effectiveness. ACS Nano **14**(10), 14134-14145 (2020). https://doi.org/10.1021/acsnano.0c06971

[S3] Chen Z, Xu C, Ma C, Ren W, Cheng HM, Lightweight and flexible graphene foam composites for high-performance electromagnetic interference shielding.

Adv. Mater. **25**(9), 1296-1300 (2013). https://doi.org/10.1002/adma.201204196

[S4] Song Q, Ye F, Yin X, Li W, Li H et al., Carbon nanotube–multilayered graphene edge plane core–shell hybrid foams for ultrahigh-performance electromagnetic-interference shielding. Adv. Mater. **29**(31), 1701583 (2017). https://doi.org/10.1002/adma.201701583

[S5] Zhao B, Hamidinejad M, Wang S, Bai P, Che R et al., Advances in electromagnetic shielding properties of composite foams. J. Mater. Chem. A **9**(14), 8896-8949 (2021). https://doi.org/10.1039/D1TA00417D

[S6] Yang W, Shao B, Liu T, Zhang Y, Huang R et al., Robust and mechanically and electrically self-healing hydrogel for efficient electromagnetic interference shielding. ACS Appl. Mater. **10**(9), 8245-8257 (2018). https://doi.org/10.1021/acsami.7b18700

[S7] Lu S, Ouyang B, Han S, Qiao F, Chen K et al., Flexible polypyrrole nanotube–polyethylene glycol–polyvinyl alcohol hydrogels for enhanced electromagnetic shielding. ACS Appl. Nano Mater. **5**(8), 11407-11413 (2022). https://doi.org/10.1021/acsanm.2c02468

[S8] Yu Z, Zhao Y, Liu J, Wang Y, Qin Y et al., Advancement in cellulose-based multifunctional high conductive PNIPaam/Ppy hydrogel/cotton composites for EMI shielding. Cellulose **29**(12), 6963-6981 (2022). https://doi.org/10.1007/s10570-022-04698-0

[S9] Xu H, Liu D, Song Y, Xie Y, Shi Z et al., Ultra-sensitive and flexible electronic skin from nanocellulose/agnws hydrogel films with highly transparent, antibacterial and electromagnetic shielding properties. Compos. Sci. Technol. **228,** 109679 (2022). https://doi.org/10.1016/j.compscitech.2022.109679

[S10] Hao M, Wang Y, Li L, Lu Q, Sun F et al., Stretchable multifunctional hydrogels for sensing electronics with effective emi shielding properties. Soft Matter **17**(40), 9057-9065 (2021). https://doi.org/10.1039/D1SM01027A

[S11] Xia B, Li T, Chen M, Wang S, Dong W. L-citrulline-modified Ti_3C_2Tx mxene nanosheets embedded in polyacrylamide/sodium alginate hydrogels for electromagnetic interference shielding. ACS Appl. Nano Mater. **5**(12), 18664-18669 (2022). https://doi.org/10.1021/acsanm.2c04437

[S12] Zhu Y, Liu J, Guo T, Wang JJ, Tang X et al., Multifunctional Ti_3C_2Tx mxene composite hydrogels with strain sensitivity toward absorption-dominated electromagnetic-interference shielding. ACS Nano **15**(1), 1465-1474 (2021). https://doi.org/10.1021/acsnano.0c08830

[S13] Wei J, Zhu C, Zeng Z, Pan F, Wan F et al., Bioinspired cellulose-integrated mxene-based hydrogels for multifunctional sensing and electromagnetic interference shielding. Inter. Mater. **1**(4), 495-506 (2022). https://doi.org/10.1002/idm2.12026

[S14] Yu Y, Yi P, Xu W, Sun X, Deng G et al., Environmentally tough and stretchable mxene organohydrogel with exceptionally enhanced electromagnetic interference shielding performances. Nano-Micro Lett. **14**(1), 77 (2022). https://doi.org/10.1007/s40820-022-00819-3

[S15] Wang J, Li Q, Li K, Sun X, Wang Y et al., Ultra-high electrical conductivity in filler-free polymeric hydrogels toward thermoelectrics and electromagnetic interference shielding. Adv. Mater. **34**(12), 2109904 (2022). https://doi.org/10.1002/adma.202109904

[S16] Liu J, McKeon L, Garcia J, Pinilla S, Barwich S et al., Additive manufacturing of Ti_3C_2-mxene-functionalized conductive polymer hydrogels for electromagnetic-interference shielding. Adv. Mater. **34**(5), 2106253 (2022). https://doi.org/10.1002/adma.202106253

[S17] Xiang M, Niu H, Qin S, Yang R, Lin W et al., Modification of graphene by polypyrrole and ionic liquids for dual-band electromagnetic interference shielding hydrogels. J. Mater. Sci. Technol. **57**(24), 10983-10996 (2022). https://doi.org/10.1007/s10853-022-07247-z
